# The Status of Environmental Electric Field Detection Technologies: Progress and Perspectives

**DOI:** 10.3390/s24175532

**Published:** 2024-08-27

**Authors:** Qingsong Liu, Zhaoqing Lan, Wei Guo, Jun Deng, Xiang Peng, Minghe Chi, Shunbo Li

**Affiliations:** 1 Electric Power Research Institute, CSG EHV Power Transmission Company, Guangzhou 510663, China; dengjun@ehv.csg.cn (J.D.); peng_x04@126.com (X.P.); 2 Joint Laboratory of DC Power Transmission Equipment and Submarine Cable Safe Operation, CSG EHV Power Transmission Company, Guangzhou 510663, China; 3 Key Laboratory of Optoelectronic Technology and Systems, Ministry of Education, College of Optoelectronic Engineering, Chongqing University, Chongqing 400044, China; zhaoqing_lan@163.com (Z.L.); 18944514605@163.com (W.G.); 4 Key Laboratory of Engineering Dielectrics and Its Application, Ministry of Education, Harbin University of Science and Technology, Harbin 150080, China; chiminghe1985@hrbust.edu.cn

**Keywords:** environmental electric field, partial discharge, NV center, electric field sensor, electro-optic effect, MEMS, NV center

## Abstract

The detection of electric fields in the environment has great importance for understanding various natural phenomena, environmental monitoring, and ensuring human safety. This review paper provides an overview of the current state-of-the-art technologies utilized for sensing electric fields in the environment, the challenges encountered, and the diverse applications of this sensing technology. The technology is divided into three categories according to the differences in the physical mechanism: the electro-optic effect-based measurement system, the MEMS-based sensor, and the newly reported quantum effect-based sensors. The principles of the underlying methods are comprehensively introduced, and the tentative applications for each type are discussed. Detailed comparisons of the three different techniques are identified and discussed with regard to the instrument, its sensitivity, and bandwidth. Additionally, the challenges faced in environmental electric field sensing, the potential solutions, and future development directions are addressed.

## 1. Introduction

The detection of electric fields is of great significance to the environment, industry, and scientific research. The existence of electrification in the atmosphere in fair weather has been recognized since 1753. At ground level on Earth, the electric field intensity is typically about 100–200 V/m in an open place in fair weather [1]. When charged thunderclouds are present nearby, the local electric field can dramatically increase to several kV/m, and lightning may also occur. Lightning is a natural disaster, which is one of the major causes of weather-related human injuries and death. In addition, the strong electric field in the air during thunderstorms and lightning affects the charging of aircraft and, consequently, safe take-off or launching [2,3]. In this regard, electrostatic sensors play a crucial role in studying atmospheric electric fields, lightning phenomena, and air ionization processes. Electric field monitoring in areas prone to fire and explosion is also important to ensure the workers’ safety and prevent economic losses. These areas include gas stations, refineries, flour mills, etc. A field mill is a useful instrument for measuring the strength of the electric field in the atmosphere, especially at airports and rocket launching bases. The main structure of a field mill has two metal electrodes. The upper one is grounded and rotates during measurement, which acts as a Faraday cage and shields the electric field underneath it. The external electric field induces a potential on the lower electrode each time it is uncovered. It disappears again when it is covered by the upper electrode. The output voltage from this oscillating signal is directly proportional to the strength of the environment’s electric field. A field mill for electric field detection is considered to be an induction method and is simple, robust, and precise on the order of V/m, which is suitable for the prediction of thunderstorms and lightning. However, it has a large volume, is difficult to integrate, and has a high cost and power dissipation. Investigating weakly charged clouds requires compact instrumentation and the ability to be carried aloft by a variety of modern airborne platforms. Harrison et al. showed that a miniature electric field meter can be implemented using computer-machined circuit boards as electrodes, simplifying the fabrication of the key mechanical components [4]. Because of its simplified equipment, small size, light mass, and low power consumption, this device opens up a range of new atmospheric charge and electric field measurements by employing meteorological balloons and remotely piloted aircraft as measurement platforms. The traditional field mills and the miniaturized electric field meter have been widely applied in atmospheric electric field detection. However, these sensors are always deployed outdoors, meaning they must withstand harsh environments like low and high temperatures, humidity, rain, etc. Therefore, the detection accuracy has to be ensured in different environments, and its lifespan has to be prolonged. The proper calibration and packaging of sensors are found to be effective ways to solve these problems.

The measurement of electric field strength also has wide application in power transmission apparatuses and grid lines for ensuring the safety, reliability, and efficiency of electrical infrastructure [5]. It is used in assessing insulation performance, identifying potential corona discharge and partial discharge activities, evaluating electromagnetic compatibility, and designing effective grounding systems. Measuring the electric field around insulators on high-voltage transmission lines can determine whether there is any defect in the insulators. The electromagnetic interference of overhead transmission lines is more complicated than underground cables. The electric field under the overhead HVDC transmission line contains two superimposed parts. One is called the nominal electric field, which is generated by the voltage on the wire. The other one is caused by the generation of space charges by a corona discharge. The superposition of these two parts forms a synthetic electric field. Accurate measurement of synthetic electric field distribution at ground level under the configuration line of the hybrid corridor is important from the point of view of environmental effect and transmission engineering [6]. Another problem that needs to be solved is partial discharge (PD), which is a highly localized microscopic electrical discharge under high-voltage stress, due to the presence of cracks, voids, and other imperfections within insulation between the two electrodes. PD inception voltage is the AC voltage at which the PD is first detected. The progressive deterioration of insulation due to PD can lead to the complete breakdown of the insulation. It may lead to catastrophic failure of a transformer and cause significant economic losses [7]. Electric field detection in a power system requires high accuracy, a large detection range, and extremely wide bandwidth since the sensors are always facing ultrahigh voltage, and AC and DC power transmission, as well as harmonics generated in the apparatus. Especially for the detection of partial discharge phenomenon, the bandwidth of the sensor must reach MHz, even GHz.

Overall, electric field strength measurement in environments, especially when highly charged sources such as thunderclouds and transformers are present, is essential in order to ensure the safety of people monitoring the working status and prevent economic losses. This review article summarizes the currently developed techniques for electric field strength measurement, including the electro-optic effect-based sensor, the MEMS sensor, and the newly developed quantum sensor, discusses the challenges, and provides recommendations for different application situations while ensuring measurement accuracy and reliability.

## 2. Electro-Optic Effect-Based Electric Field Measurement

The electro-optic effect (EO effect) is the phenomenon whereby the refractive index of a material can be tuned with an external electric field [8]. One of the most commonly known electro-optic phenomena is the Pockels effect [9], which occurs in non-centrosymmetric crystals or nonlinear materials. For the Pockels materials, the refractive index change is proportional to the external electric field strength, as shown in Equation (1). A higher Pockels coefficient will result in a higher sensitivity during measurement. Typical materials exhibiting the Pockels effect include Potassium dihydrogen phosphate (KDP), Ammonium dihydrogen phosphate (ADP), Lithium niobate (LiNbO_3_), Lithium tantalate (LiTaO_3_), Potassium titanyl phosphate (KTP), Barium titanate (BaTiO_3_), etc.

Unlike these non-centrosymmetric crystals, there are other materials with centrosymmetric atom/molecule structures exhibiting a nonlinear response of the material’s polarization to an external electric field. This is called the Kerr effect, named after John Kerr, who first observed it in 1875. When an electric field is present in the environment, it induces a polarization in the Kerr material, causing the atoms or molecules to slightly shift from their equilibrium positions. This induced polarization modifies the local electric field experienced by the electrons in the material, leading to a change in the refractive index [10]. The Kerr effect is observed in various materials, including gases, liquids, and solids. However, it is most pronounced in materials with high optical nonlinearity, such as certain organic molecules like carbon disulfide and nitrobenzene, and semiconductors such as gallium arsenide and silicon. In the linear Kerr effect, the change in the refractive index of a material is directly proportional to the square of the electric field strength applied to it, as illustrated in Equation (2).

(1) Δn=aE

(2) $$\Delta n=bE^{2}$$

where Δn is the change in the refractive index, E is the external electric field in the environment, a is the Pockels coefficient, and b is the Kerr coefficient.

**Figure 1:**
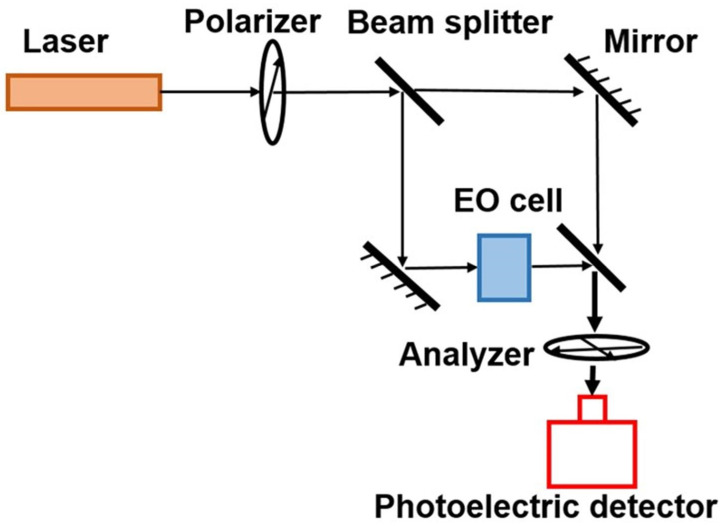
Schematic diagram of the mechanism of the electro-optic effect-based electric field sensor.

Determining electric field strength in the environment using the electro-optic effect is actually achieved by measuring the refractive index of the sensing material. Therefore, a typical Mach–Zehnder interferometer is commonly used for the measurement. As shown in Figure 1, the setup includes a few modules including the laser light source, the polarizer, the beam splitter, the mirror, the EO cell, the analyzer, and the photoelectric detector. The sensing material is placed in the EO cell, and the change in the refractive index will cause a change in the interference pattern, which is then detected by the photoelectric detector. The traditional Mach–Zehnder interferometer uses two arms for interference measurement, one has the EO cell, and the other one does not. It has stringent specifications for each component in the setup, as well as requirements for the stability of the environment, such as the vibration and flatness levels. To overcome these issues and make it applicable for on-site measurement, the optical fiber-based single optical path setup was developed [11]. The light is emitted from a single-mode fiber and then passes perpendicularly to the linear polarizer in parallel beams after the collimator. The polarization direction of the polarizer is fixed at a 45° angle with the spindle of the LiNbO_3_ crystal, so the linear polarized light will be decomposed into ordinary light (“o light”) and extraordinary light (“e light”) in the incident plane of the crystal, due to birefringence. Because of the influence of the external electric field, “o light” and “e light” have different refractive indices, which produces a phase delay in the propagation process. The output beam of the crystal is decomposed to extract two components with the same polarization direction after passing through the polarizer. The two components interfere with each other, the result of which is affected by the phase delay. The interference light intensity is converted into an electrical signal with a photoelectric converter and is quantized by an oscilloscope.

**Figure 2:**
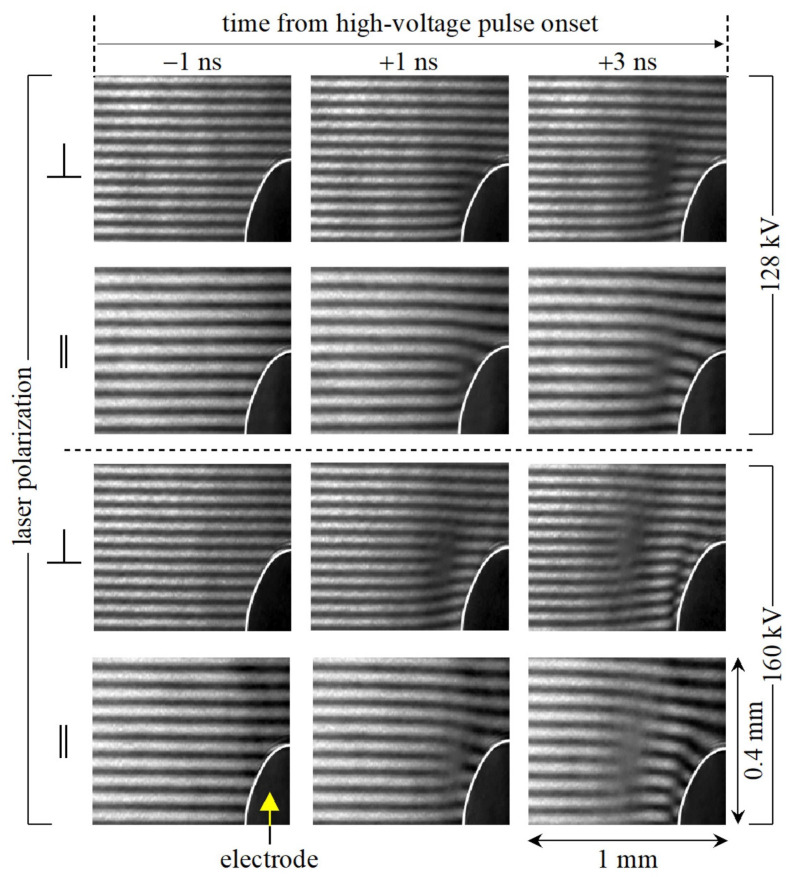
The time series of the interference fringes under different external electric fields [15]. This figure is reused under a Creative Commons license.

Compared with traditional electric field sensors, an electro-optic sensor has the advantages of high spatial resolution, wide frequency bandwidth, and little interference with the original field [12]. Therefore, it is widely used in the measurement of electric fields or transient voltages, such as the electric field of long air gap discharges and lightning propagation [13], and transient voltages in electric power systems [14]. Recently, Hoffer et al. reported the detection of a transient electric field with high spatiotemporal resolution [15]. The phase shifts caused by the Kerr effect are characterized by interference fringe shifts using a Mach–Zehnder interferometer with a picosecond laser pulse as the source and the camera as the detector. As shown in Figure 2, the interference fringes in the imaging area of 1 mm × 0.4 mm have an obvious shift after turning on the high-voltage pulse. The different directions and strengths of the electric field cause the change in the interference pattern near the electrode. This image-based detection method converts the change of the refractive index to the distinct interference fringes, which is more obvious than the traditional Mach–Zehnder interferometer shown in Figure 1. The spatial resolution is about 1 μm, and the temporal resolution depends on the width of the laser, which is 35 ps. This ultrafast detection method is highly valued in partial discharge analysis.

There are challenges faced in electric voltage or electric field measurement based on the EO effect, such as temperature sensitivity, polarization alignment, and susceptibility to optical noise. These problems have to be overcome in order to improve the sensitivity of the EO effect-based sensor. Li et al. developed a digital closed-loop detection technique based on square wave modulation and step wave feedback for the detection of AC and DC electric fields using the Pockels effect [16]. This design improved the stability of the sensor and reduced the noise. It can measure high step voltage (19.5 kV) applied to the Pockels crystal with dimensions of 8 × 15 × 4 mm. The accuracies are 0.2% and 0.5% for AC and DC signals, respectively, and the bandwidth is up to 24.5 kHz. To solve the thermal instability of LiNbO_3_ due to the thermo-optic effect, Wang et al. coated a thin layer of TiO_2_ film with a negative thermo-optic coefficient for compensation [17]. The temperature dependence was reduced to about one-tenth of the initial value after optimization of the thickness of the TiO_2_. Qi et al. applied the Kerr effect for the measurement of an electric field [18]. They developed the AC voltage modulation approach to reduce the measurement errors caused by the edge effect, the non-ideal characteristics of the optical devices (e.g., the laser source), and the system noise. This sensor could accurately measure a DC electric field of 20 V/m, and the error is smaller than 5.4%. To measure the intensive transient electric field, Zeng designed a miniaturized EO effect-based sensor with a dipole antenna [19]. The results showed the measurement of the electric field could reach up to 10 kV/cm with an error of less than 5%, and the bandwidth could be higher than 100 MHz.

As listed in Table 1, the EO effect-based voltage or electric field sensor is relatively mature and has already been applied in electrical power systems, plasma physics, and high-energy physics experiments for AC and DC electric field measurements. It shows superior characteristics in detection range and bandwidth. The sensitivity of this type of sensor is influenced by the EO coefficient, optical path in the EO materials, thermal fluctuations, optical alignment, and background noise. Shielding, filtering, compensation, and feedback techniques can help to improve the signal quality and sensitivity of the sensor. The future development of effect-based sensors is focused on miniaturization, novel EO materials with large EO coefficients, and further improvement in stability.

## 3. MEMS-Based Electric Field Measurement

**Figure 3:**
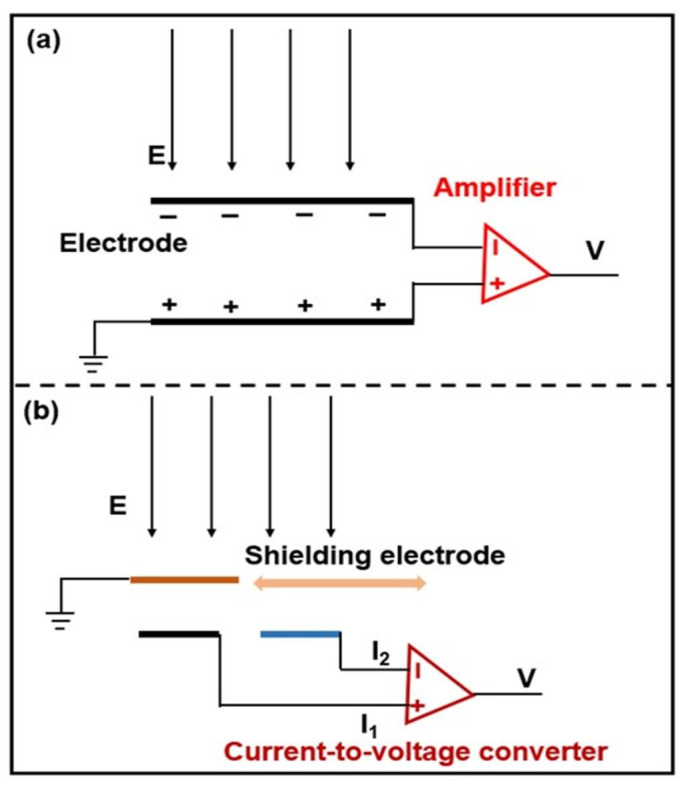
Schematic diagram of the working principle of a MEMS-based electric field sensor with static electrodes (**a**) and mobile shielding electrode (**b**).

When conducting material is present in the external electric field, there are net electrical charges generated. This is the main principle of the field mill used for detecting electric fields induced by clouds in the air. However, the whole setup is still too bulky, and the sensitivity is not enough for local electric field measurement. The microelectromechanical system (MEMS) is a novel technology that applies microfabrication techniques to fabricate small structures for sensors and actuators. MEMS technology has recently been applied to sensitive electric field measurement [20,21]. The basic structure for a MEMS electric field sensor is shown in Figure 3a. Put simply, two parallel plates and the intermedia form a capacitor, and one of the plates is grounded to work as the reference potential. There are induced charges on the two parallel electrodes when they are placed in an electric field, and the quantity of the charges is determined by the strength of the external electric field. The voltage between the two electrodes can be measured, and the sensitivity is enhanced using an amplifier. Then, the local electric field can be accurately quantified after calibration.

The induced charges are usually unstable and are influenced by the nearby media, humidity, and electromagnetic environment. To overcome these interferences, a mobile shielding structure with a fixed frequency is designed. As shown in Figure 3b, there is an induced current generated during the lateral movement of the shielding structure between the two adjacent electrodes. The electric field strength can then be obtained according to the induced current, which is measured using a current-to-voltage converter. The calibration between the electric field strength and the output voltage is also required. The generation of the motion of the shielding structure can be achieved by various methods, such as electrostatic excitation [22], thermal excitation [23,24], and piezoelectric effect excitation [25].

**Figure 4:**
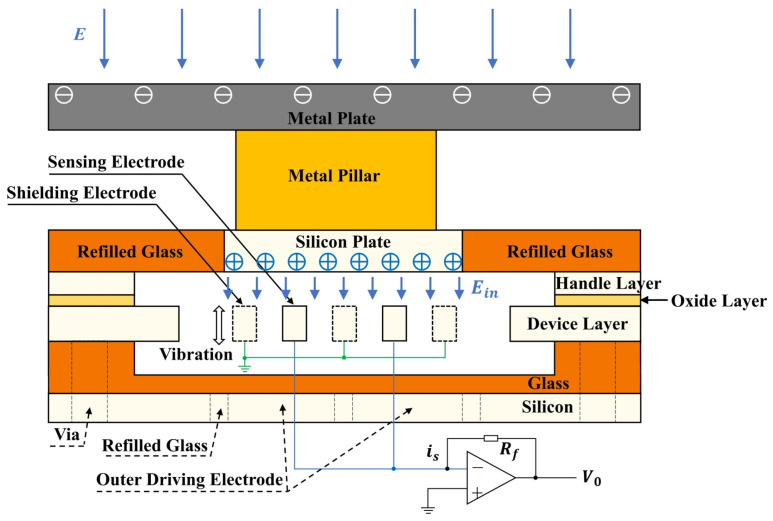
The working principle of the vertically vibrating structure and sensor package [27]. This figure is reused under a Creative Commons license.

The MEMS-based electric field sensor has the advantages of small size, low power consumption, high sensitivity, suitable for mass production, and low cost. It has been widely applied to air electric field sensing for lightning hazard warning, DC and AC electric field characterization near power transmission grids, and environmental electric field monitoring in aerospace. In 2008, Bahreyni et al. from the University of Manitoba designed an E-sensor with a fishbone thermal drive structure [23]. The designed sensor has a resonance frequency of 3892.2 Hz. The test electric field range is 0–5 kV/m, and the resolution of the sensor can reach 42 V/m. To enhance the sensitivity for electric field measurement, a probe with a size of 36 mm × 17 mm is mounted separately from the signal processing circuit. Both finite element simulation and experimental results prove that this sensor can detect an electric field as small as 5 V/m [26]. Recently, Gao et al. reported a vertical resonant MEMS electric field sensor to improve sensitivity [27]. As shown in Figure 4, the overall structure is composed of a metal plate, a metal pillar, and a microsensor. The metal plate is a metal coating on the printed circuit board, which is electrically connected to the silicon plate through the metal pillar. The metal pillar lifts the microsensor to a certain height to reduce the coupling capacitance between the metal plate and the handle layer. The external electric field is concentrated and transmitted to the silicon plate below through a metal plate and a metal pillar. The sensitivity is enhanced due to the reduction in coupling capacitance between the silicon plate and the packaging structure, as well as vertical resonance rather than parallel resonance. For detecting intense electric field applications, Yang reported a MEMS electric field sensor with a protection package [28]. This sensor has comb-shaped electrodes as sensing electrodes, and a grounded movable shutter electrode is used to induce the alternating current. The minimum detectable electric field of the sensor is 10 V/m, with an uncertainty of 0.67% in the range of 0~50 kV/m, and its power consumption is only 0.62 w. Measurement of electric fields near high-voltage direct-current (HVDC) transmission lines is essential for the safety and reliability of power systems. However, air breakdown and corona discharge in the vicinity of HVDC lines create ion flows that induce a charging effect on measurement equipment, causing significant measurement errors or even destruction of the sensor. Ma et al. from Tsinghua University proposed an E-sensor based on MEMS that can be used in high-voltage DC systems [29]. Two identical shielding chambers were designed to prevent the ion flows from accumulating on the MEMS sensors, which are located inside these chambers. The measurement results show that the MEMS sensors have a sensitivity of 10.22 kV/m and a good linearity of 0.99942, in the range of 0∼44 kV/m. The measurement error for DC synthetic fields coupled with ion flows is about 5%. These preliminary results validate the function of the MEMS sensor with protection chambers in measuring DC synthetic fields coupled with ion flows in the air.

Due to its simple system and extremely miniaturized size, the MEMS-based electric field sensor is the best candidate for three-dimensional electric field measurement. Ling et al. reported an electric field sensor with three orthogonal axes in a single chip [30]. The whole size is only about 11 mm × 11 mm for the main part of the sensor. Each axis is used to measure electrostatic field components, and the sensing and shielding electrodes are both arranged in each element. Their experimental results show that the MEMS sensor has a detection range of 0~50 kV/m, with linearity errors within 5.5% and measurement errors less than 14.0%.

As listed in Table 2, the MEMS electric field sensor, which utilizes the principles of electrostatics to measure an electric field or voltage, is a promising technology with widespread applications due to its compact size and low power consumption. The miniaturization properties make MEMS-based sensors suitable for integration into small-scale devices and systems where space is limited. They have very good sensitivity compared to EO effect-based sensors and can be used to measure both weak and strong electric field strengths. Due to the restriction of the resonant frequency of the shielding structure, the bandwidth of the MEMS electric field sensor cannot be used in AC electric field measurement at high frequency. It also faces some challenges in its lifespan due to how easily the delicate microstructures can be damaged in harsh environments. Minimizing environmental disturbance through proper sensor packaging, shielding, and isolation techniques can help maintain sensitivity under various conditions.

## 4. Quantum Effect-Based Electric Field Measurement

**Figure 5:**
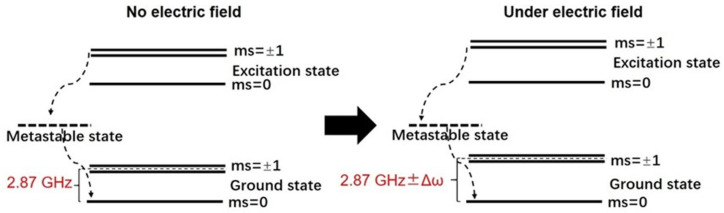
Schematic diagram of the working principle of the NV center-based electric field sensor.

With the rapid developments in quantum science and technology, quantum sensing systems like cold Rydberg atoms [31] and NV (Nitrogen-Vacancy) centers [32] have recently been applied to electric field measurement. Due to the large size of the system, the cold Rydberg atom is more popular in scientific research than in real applications. In contrast, NV centers have been used in the measurement of environmental electric fields due to the unique advantages of stable quantum states and a simple optical readout at room temperature. The NV center is a point defect in a diamond crystal, which contains a substitution N atom directly connected to a vacancy with C_v3_ symmetry [33]. The negatively charged NV center (NV^−^) is optically active and is the most popular material for quantum sensing. An optical setup that includes components such as a laser, optical fibers, lenses, and photodetectors is used to manipulate and read out the spin states of the NV centers. For the NV center, both ground and excited states show zero-field splitting, which results in degeneracy between the ms = 0 (|0〉) and ms = ±1(|±1〉) spin states. Due to its unique energy level structure, as shown in Figure 5, optical excitation with a wavelength of 532 nm can achieve both spin initialization and spin readout. For the |0〉 excitation state, it mainly relaxes to the ground state by emitting red fluorescence (637 nm). The ratio of relaxation through the metastable state (the dashed line in Figure 5) can generally be ignored. On the contrary, the |±1〉 excitation states have a large ratio of relaxing via a non-radiative transition that involves the metastable state. Through the metastable state, the spin preferentially relaxes to the |0〉 ground state and a negligibly small amount to the |±1〉 ground state. Therefore, the system can be initialized through optical pumping using a 532 nm laser. In addition, the effective brightness of the ms = ±1 is about 30% lower than the ms = 0 state. Therefore, the spin states can be optically read out by differentiating the fluorescent intensity. Usually, this is achieved by the optically detected magnetic resonance (ODMR) measurement. Firstly, it has to be noted that the energy gap between the ms = 0 and ms = ±1 states corresponds to microwave energy, with an RF frequency of 2.87 GHz. When the 532 nm laser is directed to the NV center, it is then initialized to the bright state (ms = 0). With the scanning of microwave frequency, there is a rapid decrease in fluorescence near 2.87 GHz due to the population redistribution between the ms = 0 and ms = ±1 states. The curve between the fluorescent intensity versus the microwave frequency is the so-called ODMR spectrum, from which the energy levels between different spin states can be accurately measured.

The NV center is very sensitive to small variations in external magnetic field, temperature, electric field, etc. Electric field or voltage measurements based on NV center technology utilize the unique properties of NV centers in diamond crystals. For electric field measurement, the Stark effect determines the variation in the energy gap between the ms = 0 and ms = ±1 states, as shown in Equation (3) [32]. It is clearly seen that the energy gap (2.87 GHz ± Δω) can be tuned under different electric fields, as shown in Figure 5, leading to the peak shift in the ODMR spectrum. Usually, the change in the ODMR frequency is determined after proper calibration.

(3) $$h\Delta \omega_{\pm }=d_{gs}^{∥}E_{z}\pm \left[F\left(B,E,\sigma \right)-F\left(B,0,\sigma \right)\right]$$

where h is the Planck constant, $\Delta \omega_{\pm }$ is the change in ODMR frequency, $d_{gs}^{∥}$ is the axial component of the ground triplet state permanent electric dipole moment, $E_{z}$ is the *z*-axis component of the applied electric field, and F is a physical quantity that varies according to the applied electric field E, applied magnetic field B, and the local static electric field σ.

**Figure 6:**
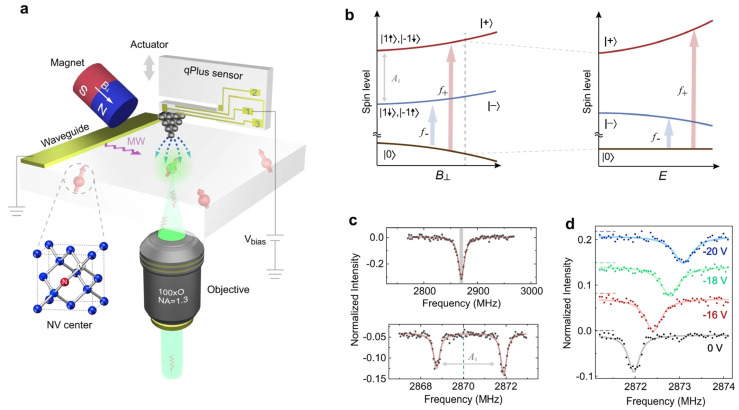
(**a**) Schematic illustration of AFM-based NV sensing: (**b**) the spin states and the corresponding energy levels under magnetic and electric fields; (**c**) continuous-wave ODMR (upper panel) and pulsed ODMR (lower panel) under zero electric fields; (**d**) pulsed ODMR spectra showing the electron spin resonance shift in f_+_ under different tip biases [35]. This figure is reused under a Creative Commons license.

The NV center-based electric field sensor has the advantages of high sensitivity and high spatial resolution. It has started to find unique applications in electric charge or electric field measurements in recent years. In 2011, Dolde et al. conducted pioneering work in the sensitive detection of an electric field generated by a single electron located about 150 nm from the NV diamond [32]. Mittiga et al. analyzed the feasibility of imaging the local charge environment of NV centers through a theoretical model [34]. The linearly polarized microwave field and the corresponding transition between the ms = 0 and ms = ±1 states are the keys to measuring the vector electric field. To measure the electric field distribution in a nanometer-sized area, Bian et al. combined the NV diamond with an atomic force microscope (AFM) [35]. As shown in Figure 6a, the AFM tip is used to position and acquire the pulsed-ODMR, and then the image of the electric field distribution is obtained by scanning. The external magnetic field is imposed by the magnet, and the waveguide is used to generate a microwave for acquiring ODMR. With the change in the external electric field, the energy levels (Figure 6b) will also be varied, corresponding to the peak shift in the ODMR (Figure 6c,d). As illustrated in Figure 6d, the ODMR peak increased from 2872 MHz to nearly 2873 MHz when the potential of −20 V is applied. The spatial resolution is about 10 nm, and the distribution of electric field strength can be clearly imaged.

Due to its high sensitivity and spatial resolution, the NV center has also been applied to biological systems with charged molecules. The charge state of an NV center is strongly influenced by its electrostatic environment. Therefore, its fluorescent signal can be tuned according to the environmental electrical potential or electric field. Karaveli et al. demonstrated a single NV can reveal a 100 mV potential swing, and multiple NVs allow for the detection of potential change as small as 20 mV [36]. Krečmarová et al. reported the use of an NV center for the detection of DNA molecules, which are charged entities in biological cells [37]. The NV center is firstly initialized from NV^−^ to NV^0^ or dark NV^+^ using a monolayer of strongly cationic charged polymer–polyethylenimine. Both NV^0^ and NV^+^ have very weak fluorescence. When negatively charged DNA molecules are immobilized on the surface of the sensor, the environmental charges are neutralized, and this restores the NV center charge state to NV^−^, which is detected using confocal photoluminescence microscopy. This NV center-based DNA sensor can detect DNA in a sample solution with a concentration as low as 100 pmol. It also has to be pointed out that the NV center is an electron spin system, indicating extreme sensitivity to a magnetic field. Therefore, reducing the interference of magnetic fields becomes the most significant issue to enhance the detection capability of NV sensors. Li et al. reported a novel way to use the continuous dynamic decoupling method [38]. Their results showed that the energy levels and the electric field strength appeared to have a simple linear relationship after removing the interference of the electric field.

The NV center has emerged as a promising tool for electric field measurement due to its unique properties, including high sensitivity, room temperature operation, robustness, nanoscale spatial resolution, and biocompatibility. The quantum state is read by the optical method. Therefore, it can minimize interference from electromagnetic environments. However, optical interference is the main source of noise. Future directions in NV diamond-based electric field sensor technology include the development of integrated sensor arrays, on-chip signal processing, remote sensing capabilities, reduction of fabrication complexity, minimization of background noise from optical and thermal fluctuations, and high cost.

## 5. Conclusions and Perspectives

In conclusion, environmental electric field sensing gives insights into studying natural phenomena, monitoring environmental parameters, and ensuring human safety. The EO effect-based sensor is mature and has a large detection range and wide frequency bandwidth. The complexity of optical path control is its main hindrance in applications. The MEMS-based sensor has ultra-high sensitivity and can easily perform 3D measurements by integrating single sensors. However, the response frequency is limited due to the vibration of the shielding electrode. The NV center-based sensor has recently been developed, and it has high spatiotemporal resolution (at the single electron level). However, the whole setup is too bulky for on-site measurement, and it has not been applied for environmental electric field detection. The miniaturization of the NV center-based system to achieve an on-chip integrated sensor with reliable operation needs further investigation. Proper calibration, reduction of noise, and enhanced stability in different environments are the common directions for these three types of electric field sensors. For the application of these sensors, sensitivity, response frequency, deployment environment, and cost are the main factors to be considered, and all of them must be improved with further research. The EO-based sensor has a large range and wide bandwidth, but the sensitivity must be improved. On the contrary, the MEMS sensor has ultrahigh sensitivity, but the bandwidth is limited. The NV center-based sensor employs a quantum effect for detection, which can not only be applied to detect electric fields but also used to detect other parameters like magnetic fields and temperature. In addition, it has the potential for miniaturization, especially when MEMS technology is used for fabrication and integration. Therefore, the NV center-based sensor is envisioned to attract more and more attention, and applications for environmental parameter detection with improved sensitivity, stability, and bandwidth will be identified.

## Figures and Tables

**Table 1:** Electric field detection based on the EO effect.

Unique Designs	Detection Range (kV/m)	Sensitivity (V/m)/Error	Applications	Reference
An integrated electro-optic E-field sensor with mono-shielding electrode	Up to 1000	N.A.	An electric field with a huge magnitude and very short rise time, e.g., lightning propagation	[13]
A noncontact approach for measurement	N.A.	N.A.	Transient voltages in power transmission lines.	[14]
A picosecond laser interferometry	Up to 100,000	N.A.	A transient electric field with high spatiotemporal resolution	[15]
A digital closed-loop detection technique based on square wave modulation and step wave feedback	0–48,000	0.2% for AC and 0.5% for DC	DC and AC electric fields with bandwidths up to 24.5 kHz	[16]
A thin layer of TiO_2_ film with a negative thermo-optic coefficient designed for compensation	N.A.	N.A.	Electric field detection with temperature variations	[17]
AC voltage modulation approach to reduce measurement errors caused by the edge effect	0–20	<5.4%	DC electric field measurement	[18]
A miniaturized EO effect-based sensor with a dipole antenna	0–1000	<105	AC electric field bandwidth higher than 100 MHz	[19]

**Table 2:** Electric field detection based on MEMS.

Unique Designs	Detection Range (kV/m)	Sensitivity (V/m)/Error	Applications	Reference
Fishbone thermal drive structure	0–5	42	Similar to electric field mills for atmospheric electric field detection	[23]
Package cap for protection from dust, rain, and low air pressure	−30–30	5	Near-ground atmospheric E-field long-term monitoring	[26]
Vertical resonant MEMS electric field sensor based on Through Glass Via technology	0–50	820	Electrostatic electric field detection	[27]
MEMS sensor with protection package, comb-shaped electrodes	0–50	10	Lightning hazard warning applications	[28]
MEMS sensor with shielding chamber	0–44	10,220	High-voltage direct-current (HVDC) transmission lines. It can prevent ion flows.	[29]
MEMS sensor with three orthogonal axes in a single chip	0–50	<14.0%	3D electric field measurement	[30]
